# First record of *Acrocyrtus* Yosii, 1959 (Collemobla, Entomobryidae) from Chinese mainland

**DOI:** 10.3897/zookeys.260.3770

**Published:** 2013-01-18

**Authors:** Guo-Liang Xu, Zhi-Xiang Pan, Feng Zhang

**Affiliations:** 1 Key Laboratory of Vegetation Restoration and Management of Degraded Ecosystems, South China Botanical Garden, Chinese Academy of Sciences, Guangzhou 510160, Guangdong Province, P. R. China; 2 School of Life Sciences, Taizhou University, Linhai 317000, Zhejiang Province, P. R. China; 3 Key Laboratory of Zoological Systematics and Evolution, Institute of Zoology, Chinese Academy of Sciences, Beijing 100101, P. R. China

**Keywords:** *A. zhujiensis*sp. n., *A. finis*sp. n., chaetotaxy, China

## Abstract

The genus *Acrocrytus* is reported from Chinese mainland for the first time, with description of two new species *Acrocyrtus zhujiensis* **sp. n.** and *Acrocyrtus finis* **sp. n.** from Zhejiang Province, East China. They can be separated from other species of this genus by colour pattern, unscaled appendages (antennae, legs and ventral tube), interocular chaetae, labial basal chaetae, bothriotrichal complex chaetae on Abd. II–IV, microchaeta a2 on Abd. II, im on Abd. III and C1p on Abd. IV. Illustrations and a table showing main differences with closest *Acrocyrtus* species are provided.

## Introduction

*Acrocyrtus* was established by Yosii 1959 as a subgenus of *Lepidocyrtus* Bourlet, 1839 for *Lepidocyrtus (Acrocyrtus) malayanus* Yosii, 1959 having pointed dental tubercle. Yoshii and Suhardjono (1989) raised it to generic level and established three subgenera (*Acrocyrtus*, *Onerocyrtus*, *Carocyrtus*) based on scales distribution of ventral tube. Christiansen and Bellinger (1991) analyzed the phylogenetic relationships among Hawaiian *Lepidocyrtus* s.l. species and questioned the reliability of dental tubercle. Later, Soto-Adames (2000) made a phylogenetic analysis of Neotropical members of the genus, disagreeing with the previous conclusion and considering that this character has phylogenetic information useful in defining Yoshii’s subgenera; he also suggested that dental tubercle should be used in combination with other characters. Considering the availability of this character in most literature descriptions and how easily it can be observed in practice, we considered that its use at generic level is relevant.

*Acrocyrtus* is characterized by the presence of conical pointed dental tubercles, rounded and finely striated scales on body and ventral side of furcula, 8+8 ommatidia (G and H smaller), 4-segmented antennae and apical bulb absent on Ant. IV, bidentate mucro with or without accessory spinelet. It is widely distributed in Southeast Asia, such as Singapore, Malay and Indonesia. So far, more than 26 species of the genus *Acrocyrtus* were described all over the world (Pan et al. 2011). Only one species *Acrocyrtus heterolepis* Yosii, 1959 was recorded from Hong Kong (Yosii 1966) and Taiwan (Yoshii 1982), China. Recently, an unidentified species assigned to “cf. *Acrocyrtus*” was also recorded from a cave in Huanjiang (Guangxi) by Deharveng et al. (2008). The two new species of *Acrocyrtus* that are studied here represent the first ones described from mainland of China.

## Materials and methods

The specimens were cleared in lactic acid, mounted under a coverslip in Marc André II solution, observed using Leica DM2500 and Nikon 80i microscopes. The photographs were taken with Nikon SM1000 microscope using a mounted Nikon DS-Fi1 camera and enhanced with Photoshop CS2. Length data were measured with NIS-Elements Documentation (Nikon). Dorsal cephalic chaetae were designated after Gisin’s system (1967), interocular chaetae after Mari-Mutt (1979, 1986), labial palp chaetae after Fjellberg (1998), labial chaetae after Gisin (1964), dorsal body chaetae after Szeptycki (1979).

Abbreviations.Th. -thoracic segment; Abd. -abdominal segment; Ant. -antennal segment; ms -specialized S-microchaeta(e); S-chaeta(e) -specialized chaeta(e) (including ms); mac -macrochaeta(e); mic -microchaeta(e).

## Taxonomy

### Acrocyrtus zhujiensis sp. n.

urn:lsid:zoobank.org:act:D8F04C9C-6364-44CF-AA8E-B5B86580D831

http://species-id.net/wiki/Acrocyrtus_zhujiensis

**Figures 1–4.**
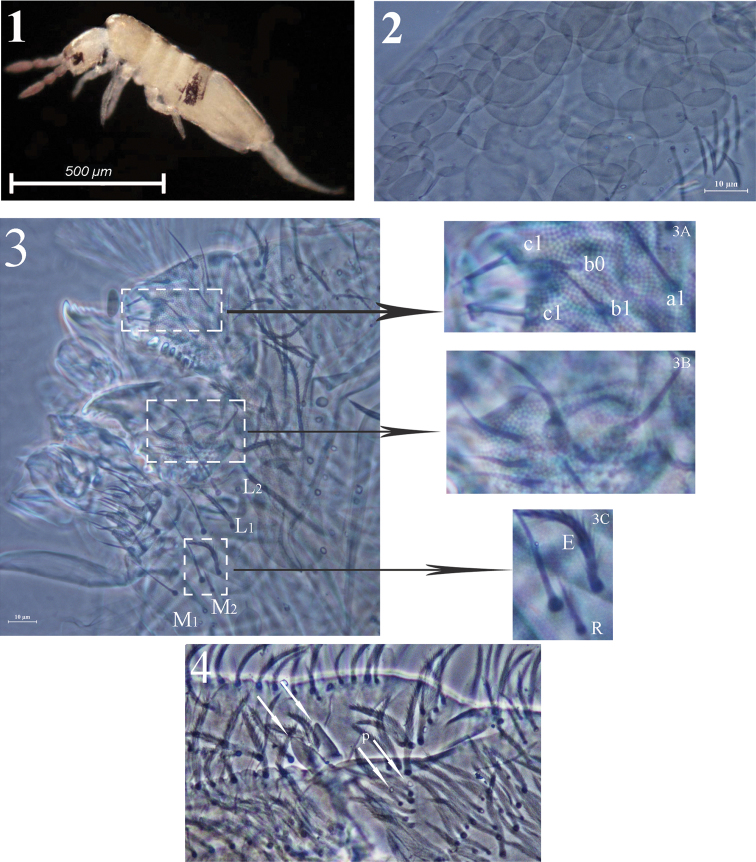
*Acrocrytus zhujiensis* sp. n. **1** colour pattern, lateral view **2** body scale **3** labium and labrum (**3A** labral intrusion **3B** maxillary outer lobe **3C** labial basal chaetae R and E) **4** apical manubrium and basal dentes.

Figs 1 [Fig F2] [Fig F3] [Fig F4] –27 , Table 1

#### Holotype.

♀ on slide, Shaoxin City, Zhuji Country, Dongbaihu, Zhejiang Province, CHINA, 29°34.18'N, 120°24.06'E, 3.X.2009, collection number S4014, collected by Zhi-Xiang Pan & Chen-Chong Si, deposited in Taizhou University.

#### Paratypes.

6 ♀ and 1 ♂ on slide and 10 in alcohol, same data as holotype. 4 paratypes (2 ♀ on slide and 2 in alcohol) deposited in School of Life Sciences, Nanjing University and others in School of Life Sciences, Taizhou University, China.

#### Etymology.

Named after the type locality.

#### Description.

Body length up to 0.93 mm.

Colour pattern. Ground colour pale yellow, with a pair of dark patches present on lateral Abd. III. Violet pigment distributed on antennae and gradually darker towards tip. Eye patches dark (Fig. 1). Scales hyaline, oval to circular (Fig. 2), present on head, body tergites, ventral side of furcula; antennae, ventral tube and legs unscaled.

**Figures 5–17. F2:**
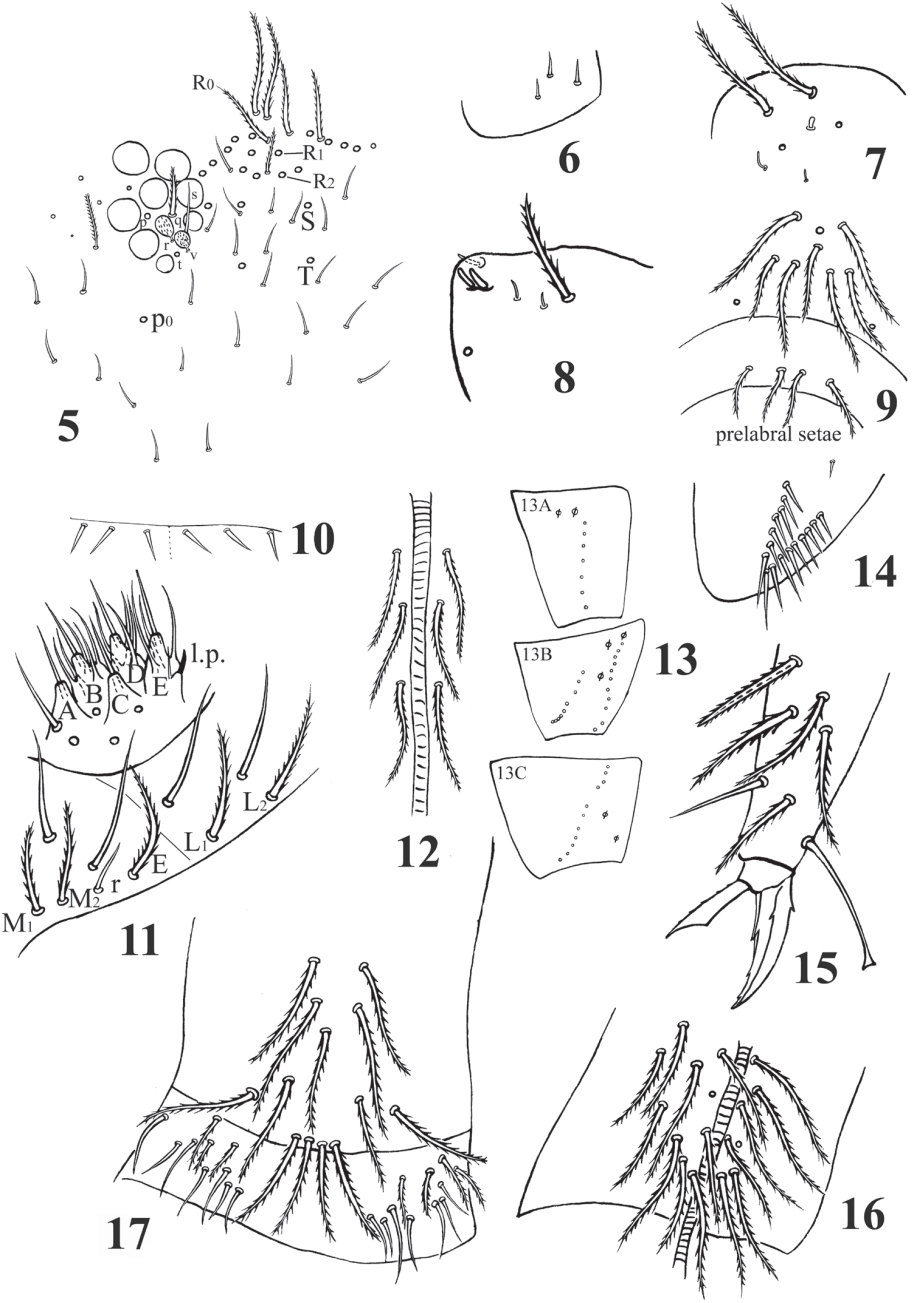
*Acrocrytus zhujiensis* sp. n. **5** head cheatotaxy **6** basal Ant. I **7** three kinds S-chaetae on Ant. II **8** Ant. III organ **9** clypeal chaetae **10** cervical chaetae **11** labial base and labial palp **12** cephalic groove **13** coxal macrochaetae (**13A** fore legs **13B** mid legs **13C** hind legs) **14** trochanteral organ **15** hind claw **16** anterior side of ventral tube **17** posterior side and lateral flap of ventral tube.

Head. Ommatidia 8+8, G and H smaller than others. Interocular chaetae as **p**, **r**, **t**, **q**, **s**, **v**; chaeta **s** smooth, chaetae **r** and **v** transformed to scales, chaetae **p**, **t** and **q** ciliate (Fig. 5). Antennae 1.5–2.4 times as long as cephalic diagonal. Antennal segmental ratio as I:II:III:IV = 1:1.3–1.4:1.2–1.9:1.9–3.1. Ant. I with 3 dorsal and 3 ventral basal spiny mic (Fig. 6). Ant. II with 4 basal tiny spiny mic, 1 distal rod-like and 12–15 normal S-chaetae (Fig. 7). Ant. III organ with 2 rod-like S-chaetae (Fig. 8). Ant. IV without apical bulb. Anterior part of head with many long, ciliate chaetae but not claviform (Fig. 5). Prelabral and labral chaetae as 4/5, 5, 4, prelabrals ciliate and others smooth, labral intrusion V-shaped, chaetae of c-row thicker than those in other rows; labral margin with 4 conical papillae (Fig. 3). Clypeal chaetae as 3-1-4, without scales between them (Fig. 9). Cervical chaetae as 16 spiny chaetae, lateral two slightly longer than others (Fig. 10). Subapical chaeta of maxillary outer lobe subequal to apical one, 3 smooth sublobal hairs on sublobal plate. Labial palp with 5 papillae as A–E, respectively with 0, 5, 0, 4, 4 guard chaetae; lateral process (l.p.) of labial palp straight, thick and blunt with tip not reaching apex of papilla E. Chaetotaxy of labial base as **M_1_M_2_REL_1_L_2_**, all ciliate, chaeta **R** shorter than others (Fig. 11). Chaetal row along labial groove with 3 ciliate chaetae, and other postlabial chaetae ciliate (Fig. 12). Mandible with 4+5 (left+right) teeth (Fig. 3).

Leg. Coxae: I, with 7 ciliate mac and 2 pseudopores; II, with 7–8 ciliate mac in the anterior row, 8–11 ciliate mac in the posterior row and 3 pseudopores; III, with 9–11 ciliate mac and 2 pseudopores (Fig. 13). Trochanteral organ with 12–17 smooth spines (1–2 inner) (Fig. 14). Unguis with 4 inner teeth (paired ones at 1/3, middle one at 2/3 and apical one at 3/4 distance from base), 2 lateral teeth (at 1/4 distance from base) and 1 outer tooth (at 1/5 distance from base). Unguiculus slender and truncate with outer edge serrate. Tenent hair clavate, subequal to inner edge of unguis in length, and slightly longer than unguiculus. A distal smooth chaeta on tibiotarsus III subequal to unguiculus in length (Fig. 15).

Ventral tube. Anterior face with 14+14 ciliate chaetae; posterior face without smooth chaeta (Fig. 16); lateral flap with 6–8 smooth and 2–4 ciliate chaetae (Fig. 17).

**Figures 18–23. F3:**
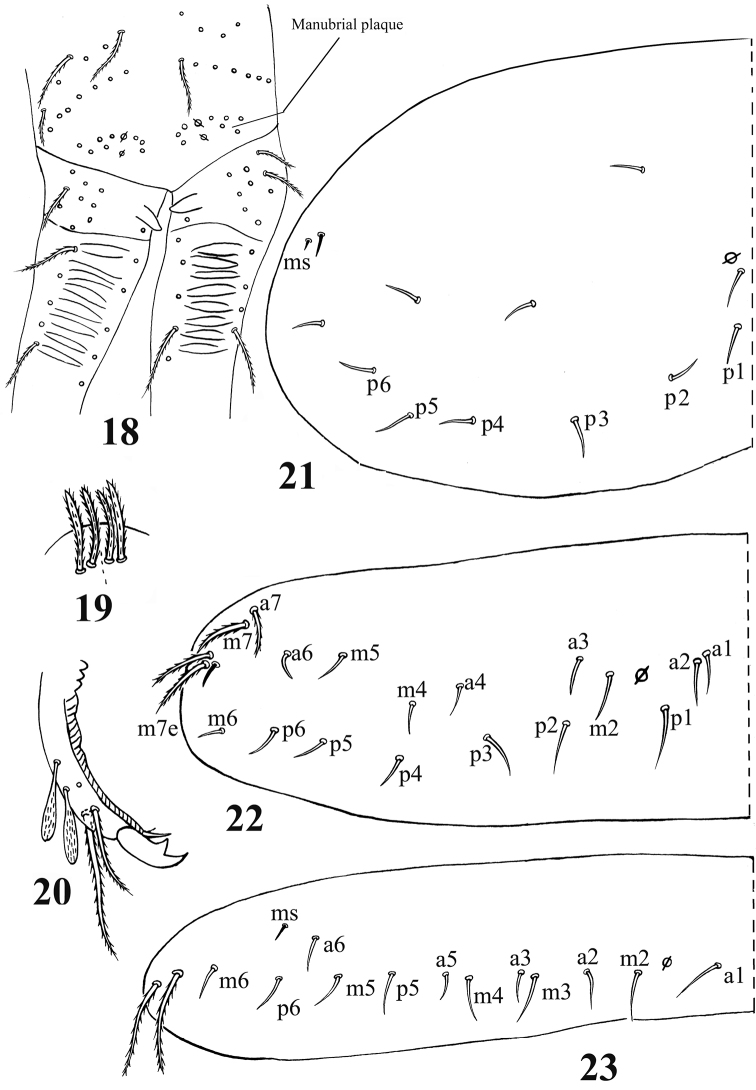
*Acrocrytus zhujiensis*sp. n. **18** distal manubrium and basal dens **19** distal part of ventral manubrium **20** mucro **21–23** dorsal chaetotaxy **21** Th. II **22** Th. III **23**. Abd. I.

Furcula. Manubrial plaque with 2–3 inner, 4–6 outer ciliate chaetae and 2 pseudopores. Dental tubercles conically pointed (Figs 4, 18). Ventral terminal manubrium with 2+2 ciliate chaetae (Fig. 19). Distal smooth part of dentes 2.1–2.5 times as long as mucro. Mucro bidentate, mucronal basal spine reaching subapical tooth with an accessory spinelet (Fig. 20).

**Figures 24–25. F4:**
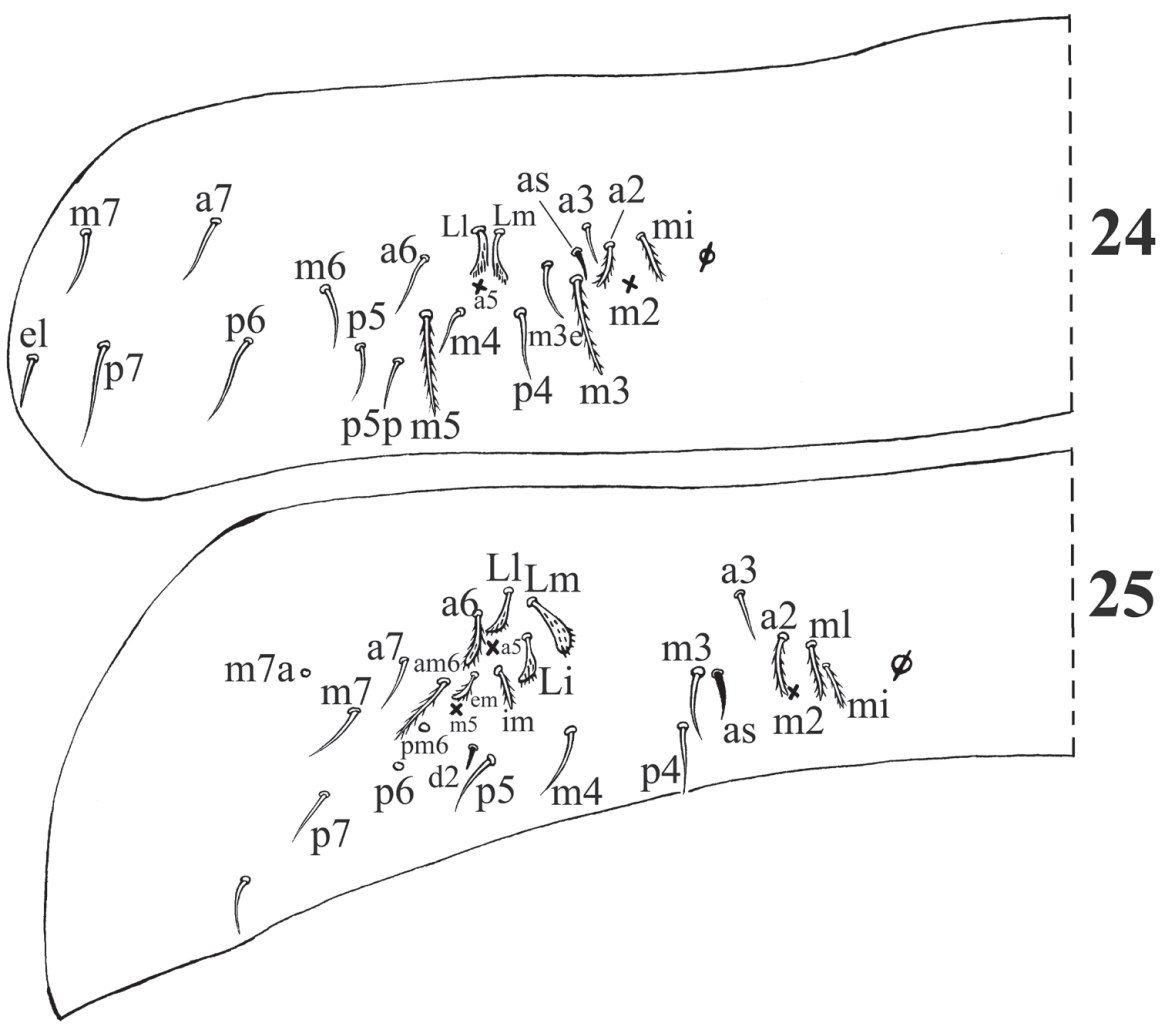
Dorsal chaetotaxy of *Acrocrytus zhujiensis* sp. n. **24** Abd. II **25** Abd. III (as: antero-lateral S-chaeta).

**Figures 26–27.**
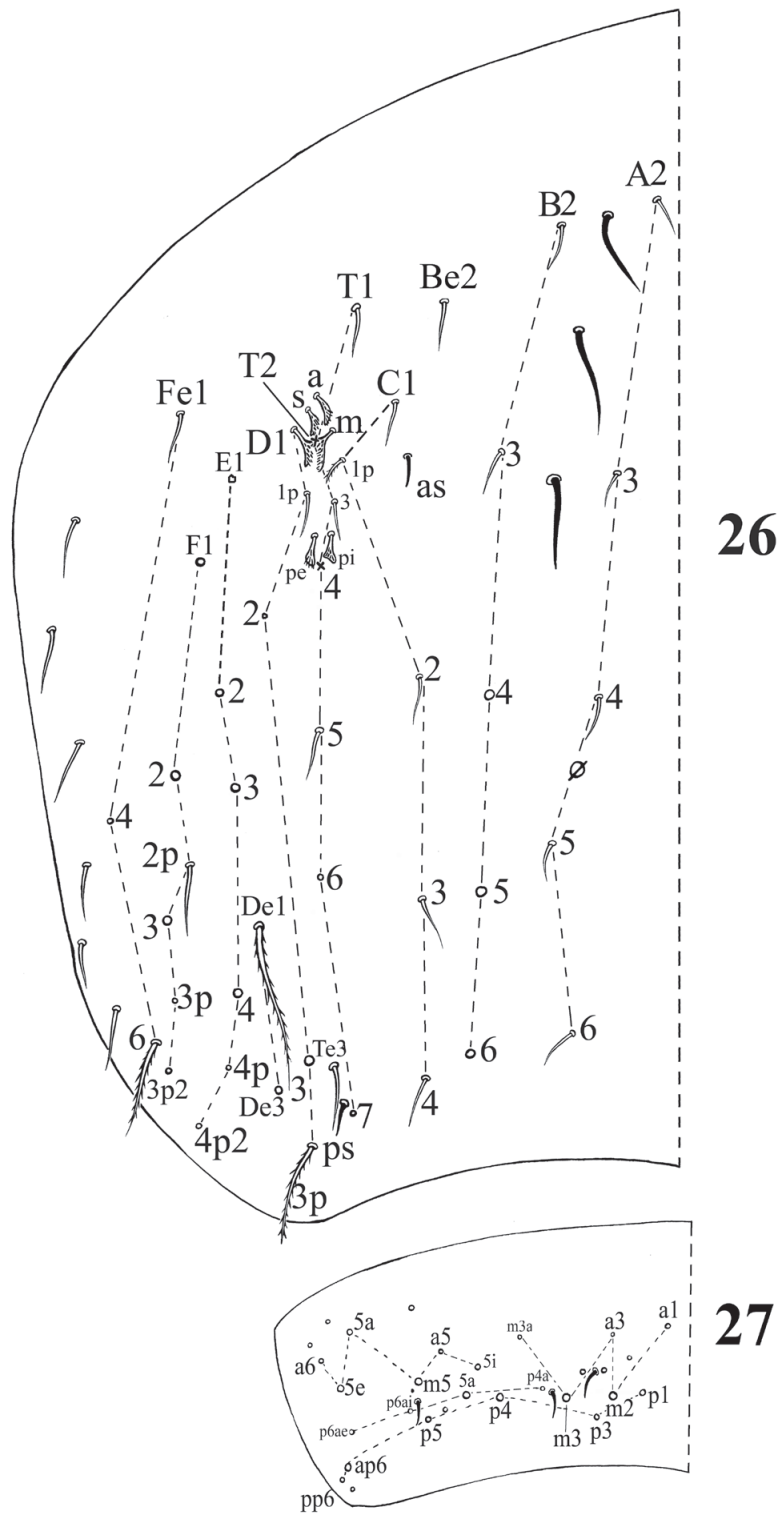
Dorsal chaetotaxy of*Acrocrytus zhujiensis* sp. n. **26** Abd. IV **27** Abd. V (ps: postero-sublateral S-chaeta).

Chaetotaxy. Dorsal cephalic mac as **R_0_R_1_R_2_STP_0_**; R_1s_ absent. Body mac as 00/0100+3, S-chaetae as 21/11253, ms as 10/10100. Th. II slightly protruded over head, with 1–2 rows of ciliate “collar” mac, 2 antero-lateral S-chaetae (ms external to another S-chaeta), 6 (**p1**–**6**) smooth mic and 5 anterior unclear homology smooth mic (Fig. 21). Th. III with 1 S-chaeta external to **m7**; 15 (**a1**–**4**, **a6**, **m2**, **m4**–**6** and **p1**–**6**) smooth mic, 3 (**a7**, **m7** and **m7e**) ciliate mac and a lateral unclear homology ciliate chaeta (Fig. 22). Abd. I with 1 ms, 12 (**a1**–**3**, **a5**–**6**, **m2**–**6** and **p5**–**6**) smooth mic and 2 lateral unclear homology ciliate chaetae (Fig. 23). Abd. II with 1 central S-chaeta (**as**), 1 (**a2**) ciliate mic, 13 (**a3**, **a6**–**7**, **m3e**, **m4**, **m6**–**7**, **p4**–**7**, **p5p** and **el**) smooth mic, 1 ciliate and slightly modified mic (**mi**), 2 (**Lm** and **Li**) ciliate, modified and fan-shaped mic, 2 (**m3** and **m5**) ciliate mac; chaetae **a2p** and **ml** absent (Fig. 24). Abd. III with 1 central S-chaeta (**as**) and 1 lateral ms, 5 (**mi**, **ml**, **a2**, **im**, **em** and **am6**; **ml** sometimes present on one side) ciliate and slightly modified mic, 4 (**Li**, **Lm**, **Ll** and **a6**) strongly modified and fan-shaped mic, 8 (**a3**, **a7**, **m3**, **m7**, **p3**–**5** and **p7**) smooth and subequal mic, 3 (**pm6**, **m7a** and **p6**) ciliate mac (Fig. 25). Abd. IV with 1 anterior (**as**) and 1 posterior (**ps**) short S-chaetae and 3 median elongate S-chaetae, 22 (**A2**–**6**, **B2**–**3**, **Be2**, **C1**–**4**, **T1**, **T3**, **T5**–**6**, **Te3**, **D2**–**3**, **D1p**, **E1** and **Fe1**) smooth mic, 2 (**C1p** and **T7**) ciliate mic, 6 (**m**, **a**, **s**, **D1**, **pi** and **pe**) ciliate, strongly modified and fan-shaped mic, 13 (**B4**–**6**, **De1**, **De3**, **D3p**, **E2**–**4**, **F2**–**3**, **F3p2**, **Fe4** and **Fe6**) ciliate mac, 4(**E4p**, **E4p2**, **F2p** and **F3p2**) mic (Fig. 26). Abd. V with 3 S-chaetae (Fig. 27).

#### Ecology.

In the leaf litter of *Cunninghamia lanceolata*, *Cinamomum camphora* along a lake.

#### Remarks.

This new species is characterized by colour pattern, clavate tenent hair, unscaled appendages (antennae, legs and ventral tube), 4 conical labral papillae, ventral tube with ciliate chaetae present on lateral flap and without smooth chaeta on posterior side, and ciliate mic **a2** on Abd. II.

It is similar to Vietnamese *Acrocyrtus baii* Nguyen, 2005 in clavate tenent hair, claw, unscaled appendages (antennae and ventral tube). However, it can be easily distinguished from it by pigment absent on Th. II–III and Abd. II (versus present), ciliate labial chaetae **EL_1_L_2_** (versus smooth), **M_1_** subequal to **M_2_** on labial base (versus **M_1_** smaller than **M_2_**), absence of smooth chaetae on posterior face of ventral tube (versus 1+1 smooth chaetae) and unscaled legs (versus scaled).

### Acrocyrtus finis sp. n.

urn:lsid:zoobank.org:act:0E689089-71E5-4FD4-AD50-3A2B23CE5595

http://species-id.net/wiki/Acrocyrtus_finis

**Figures 28–30.**
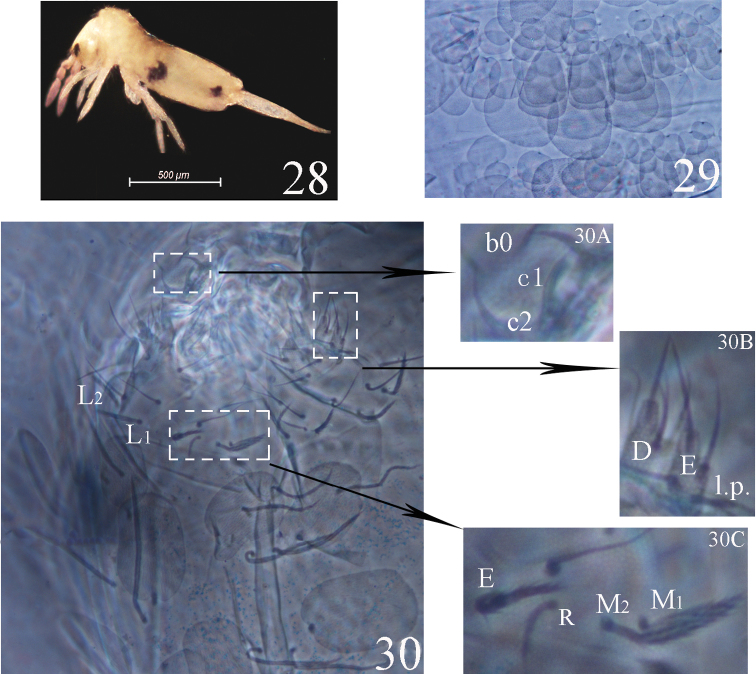
*Acrocrytus finis*sp. n. **28** colour pattern, lateral view **29** body scale **30** labium and labrum (**30A** labral intrusion **30B** labial papillae D, E and l.p. **30C** labial base).

Figs 28 [Fig F7] [Fig F8] –37 , Table 1

#### Holotype.

1 ♀ on slide, Taizhou City, Dalei Mountain, Zhejiang Province, CHINA, 29°02.25'N, 120°53.03'E, 25.X.2009, collection number S4023, collected by Zhi-Xiang Pan, deposited in Taizhou University.

#### Paratypes.

11 ♀ on slide and 15 in alcohol, same data as holotype. 4 paratypes (2 ♀ on slide and 2 in alcohol) deposited in School of Life Sciences, Nanjing University and others in School of Life Sciences, Taizhou University, China.

#### Etymology.

Named after the type locality, which is the border (latin word “finis”) of the three adjacent cities.

#### Description.

Body length up to 1.2 mm.

Colour pattern. Ground colour from yellow to slightly brown, a pair of dark lateral patches of Abd. III and a pair of dark postero-lateral patches of Abd. IV, slightly violet pigment distributed on antennae and gradually darker towards tip, eye patches dark (Fig. 28). Scales hyaline, oval to circular (Fig. 29), present on head, body tergites and ventral side of furcula, and absent on antennae, legs and ventral tube.

Head. Ommatidia 8+8, G and H smaller than others, interocular chaetae as **p**, **r**, **t**, **q**, **s**, **v**; chaeta **s** smooth, chaetae **p**, **t**, **q** ciliate, chaetae **r** and **v** transformed to scales. Antennae 1.4–2.0 times as long as cephalic diagonal. Antennal segmental ratio as I:II:III:IV = 1:1.3–1.9:1.5–2.5:2.5–5.0. Ant. I with 3 dorsal and 3 ventral basal spiny chaetae. Ant. II with 4 basal tiny spines, 11–14 short and 1 distal rod-like S-chaetae. Ant. III organ with 2 rod-like S-chaetae. Ant. IV without apical bulb. Anterior part of head with many ciliate and long, but not claviform chaetae. Cervical with 16 spiny chaetae, all subequal in length. Prelabral and labral chaetae as 4/5, 5, 4, prelabrals ciliate and others smooth, chaetae of c-row thicker than other row chaetae; labral intrusion V-shape; labral margin with 4 papillae. Clypeus without scales. Subapical chaeta of the maxillary outer lobe subequal to apical chaeta, 3 smooth sublobal hairs on sublobular plate. Labial palp with five papillae as A–E, respectively with 0, 5, 0, 4, 4 guard chaetae; lateral process of labial palp straight, thick with tip not reaching apex of papilla E. Chaetotaxy of labial base as **M_1_M_2_REL_1_L_2_**, all ciliate, chaeta **r** shorter than others. Chaetal row along labial groove with 3 ciliate chaetae, and other postlabial chaetae ciliate. Mandible with 4+5 (left+right) teeth (Fig. 30).

Leg. Coxae: I, with 5–7 ciliate mac and 2 pseudopores; II, with 6–7 ciliate mac in the anterior row, 7–9 ciliate mac in the posterior row and 3 pseudopores; III, with 6–7+3 ciliate mac and 2 pseudopores. Trochanteral organ with 10–14 smooth spiny chaetae. Unguis with 1 outer (at 1/5 distance from base), 2 lateral (at 1/4 distance from base) and 4 inner teeth (paired ones at 1/3, middle one at 2/3 and apical one at 3/4 distance from base to apical inner unguis), all tiny. Unguiculus slender and truncate with outer edge slightly serrate. Tenent hair clavate, subequal to inner margin of unguis, and slightly longer than unguiculus. Supraempodial chaeta subequal to unguiculus.

Ventral tube. Anterior face with 9–15 larger ciliate chaetae; posterior face without smooth chaetae; lateral flap with 6–7 smooth and 2–3 ciliate chaetae.

Furcula. Manubrial plaque with 2–3 inner, 4–6 outer ciliate chaetae and 2 pseudopores, ventral manubrium with 2+2 ciliate terminal chaetae. Dental tubercles conically pointed. Distal smooth part of dentes 1.5–2.0 times as long as mucro. Mucro bidentate, mucronal basal spine reaching subapical tooth with two accessory spinelets.

**Figures 31–33. F7:**
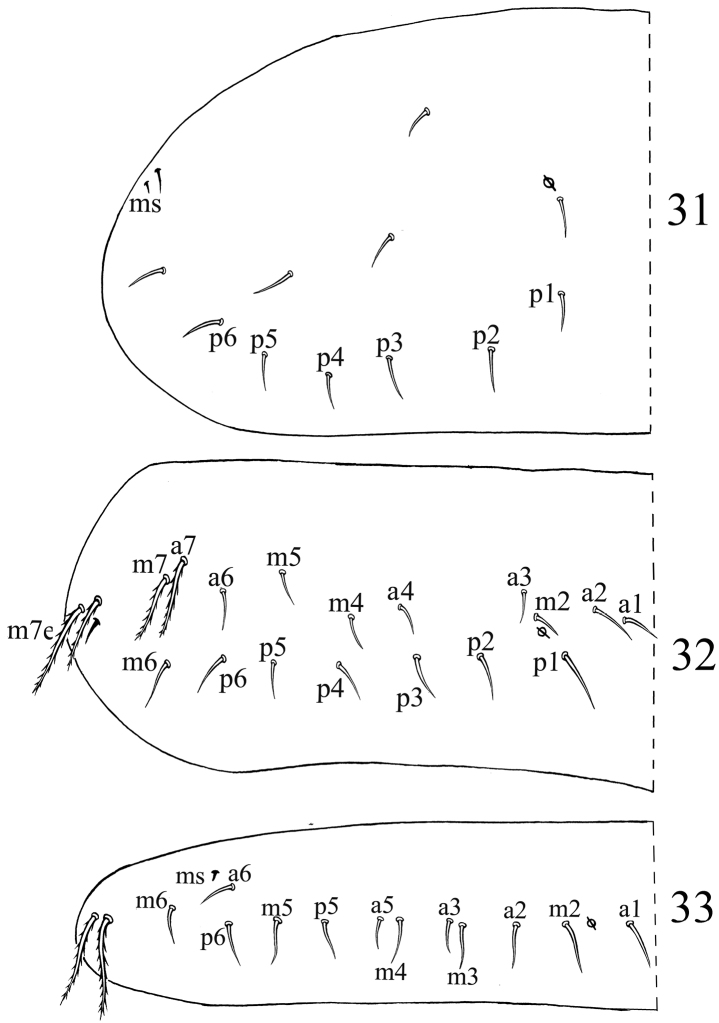
Dorsal chaetotaxyof *Acrocrytus finis* sp. n. **31** Th. II **32** Th. III **33** Abd. I.

**Figures 34–35. F8:**
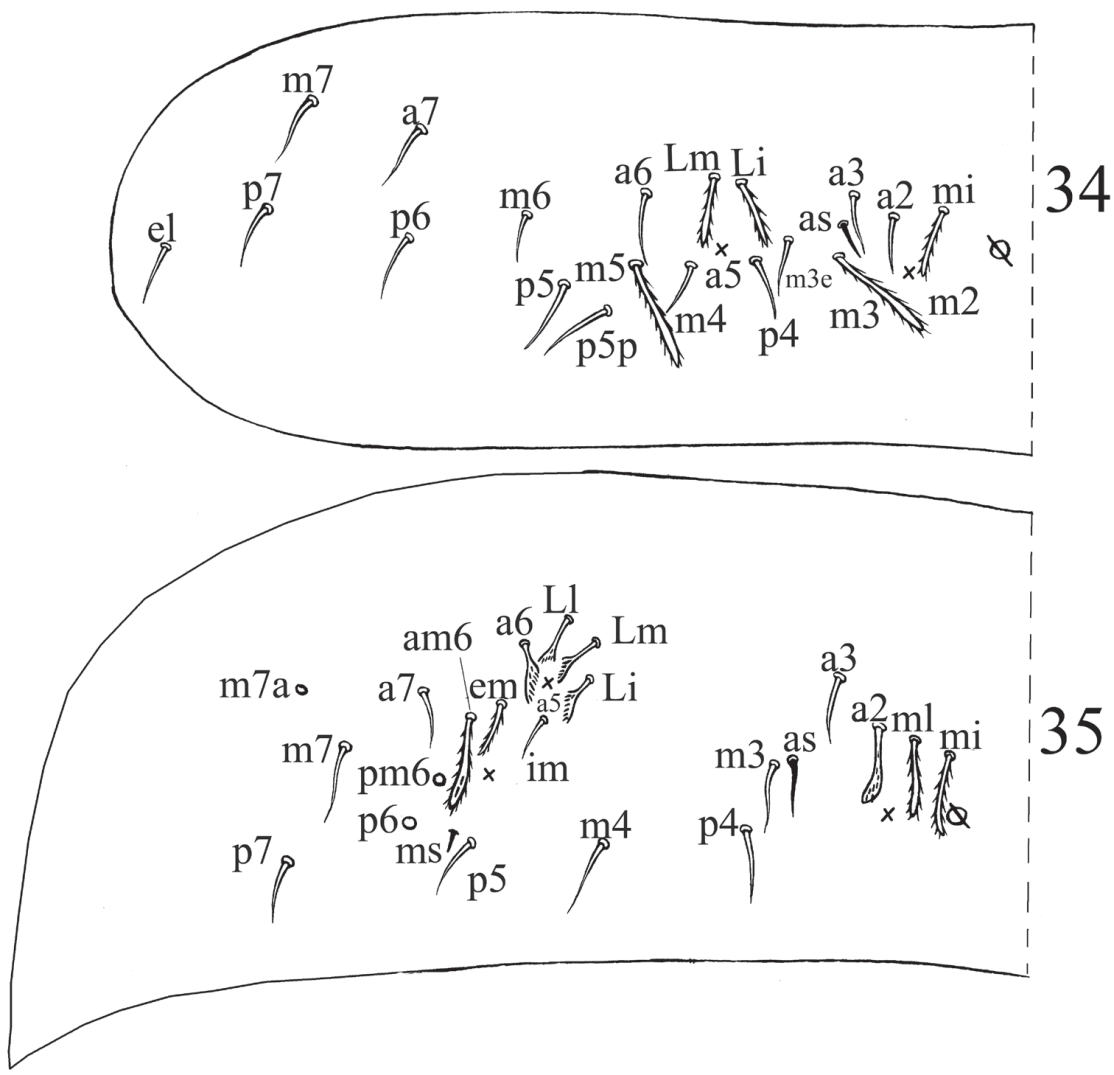
Dorsal chaetotaxyof *Acrocrytus finis* sp. n. **34** Abd. II **35** Abd. III.

**Figures 36–37.**
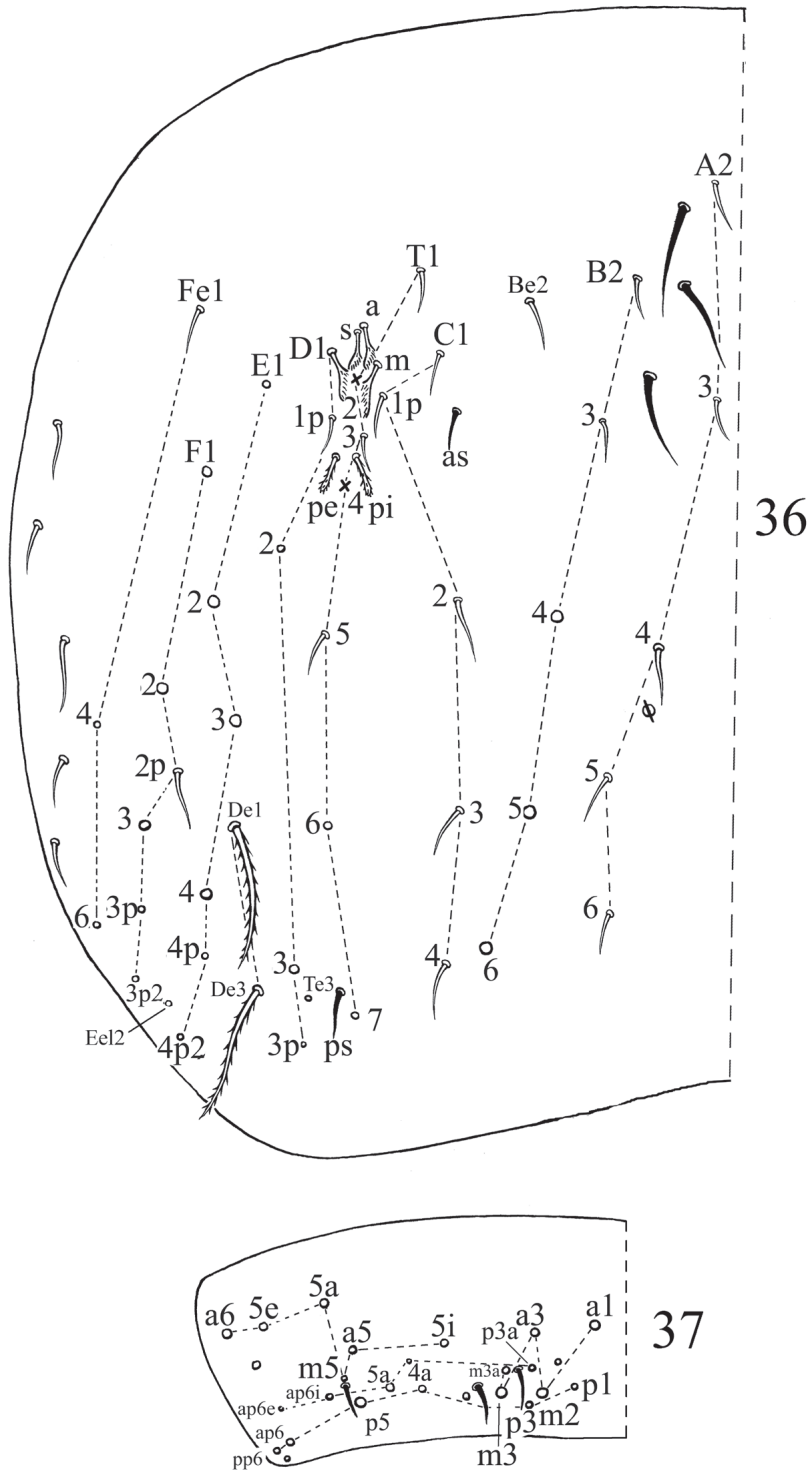
Dorsal chaetotaxyof *Acrocrytus finis* sp. n. **36** Abd. IV **37** Abd. V.

Chaetotaxy. Dorsal cephalic mac as **R_0_R_1_R_2_TS**, **P_0_** sometime absent. Body mac as 00/0100+3, S-chaetae as 21/11253, ms as 10/10100. Th. II slightly protruded over head, with 2 antero-lateral S-chaetae (ms postero-external to another one), 6 (**p1**–**6**) smooth and subequal mic and 5 anterior smooth mic of unclear homology (Fig. 31). Th. III with 1 S-chaetae external to **m7**, 15 (**a1**–**4**, **a6**, **m2**, **m4**–**6** and **p1**–**6**) smooth mic, 3 (**a7**, **m7** and **m7e**) mac and one other mac of unclear homology (Fig. 32). Abd. I with 1 ms external to **a6**, 12 (**a1**–**3**, **a5**–**6**, **m2**–**6** and **p5**–**6**) smooth mic and 2 lateral ciliate mac of unclear homology (Fig. 33). Abd. II with 1 central S-chaetae (**as**), 1 (**mi**) ciliate and blunt mic, 14 (**a2**–**3**, **a6**–**7**, **m3e**, **m4**, **m6**–**7**, **p4**–**5**, **p5p**, **p6**–**7** and **el**) smooth and subequal mic, 2 (**Lm** and **Li**) ciliate and slightly expanded mic, 2 (**m3** and **m5**) ciliate mac with tip expanded, chaetae **a2p** and **ml** absent (Fig. 34). Abd. III with 1 central S-chaeta (**as**) and 1 lateral ms, 5 (**a2**, **mi**, **ml**, **em** and **am6**) ciliate mic with tip expanded, 4 (**Li**, **Lm**, **Ll** and **a6**) ciliate modified and fan-shaped mic, 9 (**im**, **a3**, **a7**, **m3**, **m7**, **p3**–**5** and **p7**) smooth mic, 3 (**pm6**, **m7a** and **p6**) ciliate mac (Fig. 35). Abd. IV with 1 anterior (**as**) and 1 posterior (**ps**) short S-chaetae and 3 elongate median S-chaetae, 22 (**A2**–**6**, **B2**, **B3**, **Be2**, **C1**–**4**, **C1p**, **T1**, **T3**, **T5**–**6**, **D1p**, **D2**–**3** and **Fe1**) smooth mic, 15 (**B4**–**6**, **T7**, **D3p, De1**, **De3**, **E1**–**4**, **F1**–**3**, **Fe4** and **Fe6**) ciliate and mac, 5 (**Te3**, **E4p**, **E4p2**, **F3p** and **F3p2**) as mic, 4 (**m**, **a**, **s** and **D1**) ciliate and strongly fan-shaped mic, 2 (**pi** and **pe**) ciliate mic with tip expanded (Fig. 36). Abd. V with 3 S-chaetae (Fig. 37). Abd. IV:Abd. III = 2.6–4.1:1.

#### Ecology.

In leaf litter of *Pinus massoniana*, *Schima superba* and *Cinamomum camphora*, in bryophyta and on farmland.

#### Remarks.

The new species is easily distinguished from other *Acrocyrtus* by 4 abdominal lateral patches, morphology of interocular chaetae **v**, **r** and **s**, cephalic mac as **R_0_R_1_R_2_ST**, 4 papillae and thicken c-row chaetae on labrum, smooth mic **a2**, **im** and **C1p** on Abd. II, Abd. III and Abd. IV, respectively, unscaled appendages (antennae, ventral tube and legs).

The species is most similar to *Acrocyrtus zhujiensis* sp. n. in cephalic chaetotaxy, labral papillae, claw, furcula, macrochaetotaxy and S-chaetotaxy. However, the two species are different in colour pattern, morphology of chaetae **a2**, **m3** and **m5** on Abd. II, **a2** and **im** on Abd. III, **C1p** on Abd. IV. Main differences between two new species are listed in Table 1.

**Table 1.** Main differences between three similar species of *Acrocyrtus*.

	***Acrocrytus zhujiensis* sp. n.**	***Acrocrytus finis* sp. n.**	***Acrocrytus baii***
Dark patches laterally on Abd. II	absent	absent	present
Dark patches postero-laterally on Abd. IV	absent	present	present
Apical bulb of Ant. IV	absent	absent	present
Number of labral papillae	4	4	0
Chaeta **M_2_** on labial base	ciliate	ciliate	smooth
Chaeta **R** on labial base	slightly ciliate	slightly ciliate	reduced
Chaetae **EL_1_L_2_** on labial base	ciliate	ciliate	smooth
Inner teeth on unguis	4	4	3
Smooth chaetae on posterior ventral tube	0+0	0+0	1+1
Chaeta **a2** on Abd. II	ciliate	smooth	?
Chaetae **m3** and **m5** on Abd. II	not expanded	expanded	?
Chaeta **a2** on Abd. III	not expanded	expanded	?
Chaeta **im** on Abd. III	ciliate	smooth	?
Chaeta **C1p** on Abd. IV	ciliate	smooth	?
Distribution	China	China	Vietnam

?: character not provided in original description

## Discussion

Dental tubercles are pointed in *Acrocyrtus* and rounded in *Ascocyrtus*. However, it is sometimes uneasy to recognize the shape of dental tubercles. Their shape maybe wrongly observed in different visual angles, pointed in lateral view but “rounded” in facial view. They need to be observed under various angles. Another interesting point is that some species of Yoshii (1982, 1989) with colour patterns similar to our species (pigment on Abd. III) were assigned to *Ascocyrtus* rather than *Acrocyrtus*. Our new species would be placed in *Ascocyrtus* due to habitus compared with those Southeast Asia taxa (Deharveng, personal communication). Actually, more Chinese species with similar patterns in our collection have pointed and relatively long dental tubercles. Since the works of Yosii, no significant advance has been made for the classification of *Lepidocyrtus* s. l. Molecular tools are expected to help discriminate Lepidocyrtinae genera in the future.

## Supplementary Material

XML Treatment for Acrocyrtus zhujiensis

XML Treatment for Acrocyrtus finis
